# 554. Bromelin-based Enzymatic Debridement in Full and Deep-partial Thickness Burns in the ICU: A Case Series

**DOI:** 10.1093/jbcr/irag033.294

**Published:** 2026-04-06

**Authors:** Kristen Kelliher, Melanie Mait, Andrew C Eksi, Monica Stevens, Rosemary E Paine, Elizabeth N Turner, Damien W Carter

**Affiliations:** MaineHealth Medical Center, Portland, ME; MaineHealth Medical Center, Portland, ME; MaineHealth Department of Surgery, Portland, ME; MaineHealth Medical Center, Portland, ME; MaineHealth Medical Center, Portland, ME; MaineHealth Medical Center, Portland, ME; MaineHealth Medical Center, Portland, ME

## Abstract

**Introduction:**

Early surgical debridement of full and deep partial-thickness burns is fundamental in burn care. Patients with large total body surface area (TBSA) burns may be too unstable for operative intervention. Surgical excision can result in removal of healthy dermis, blood loss, and prolonged operative time, all of which are detrimental to critically ill burn patients. Conversely, delayed excision predisposes patients to infections, prolonged burn shock, and poor cosmesis. Traditionally, enzymatic debridement has been reserved for smaller TBSA, non-critically ill patients, or within the first 72 hours of hospital presentation. Our burn center demonstrated bromelin-based enzymatic debridement (BBED) for critically ill patients as an effective and safe alternative to surgical excision.

**Methods:**

We report a single institution experience with 4 patients admitted to the Surgical Intensive Care Unit who underwent early topical application of BBED instead of surgical excision of burns. Median age was 39 (34-67 years) and median TBSA was 24% (22-46%). All four patients had thermal burns from house fires with concomitant inhalation injuries (Grades 1 or 2) and were deemed too medically unstable to undergo early surgical excision. All patients underwent eventual split thickness skin grafting (STSG). One patient underwent additional operative debridement and allografting of their burns prior to autografting, and two patients underwent successful BBED while on systemic anticoagulation (AC).

**Results:**

All four patients who initially underwent BBED later underwent split-thickness skin autografting to portions of the treatment area. There were no identifiable adverse effects related to enzymatic debridement. Two patients who were on systemic anticoagulation tolerated BBED without bleeding complications.

**Conclusions:**

Our case series shows BBED is safe in critically ill patients and well tolerated.

**Applicability of Research to Practice:**

To our knowledge, this is the first reported application of enzymatic debridement of burns in critically ill patients in a delayed (> 72 hours from burn) manner and can serve as an example for other institutions. More studies are needed to assess a large scale of patients and across institutions who undergo BBED over surgical debridement.

**Funding for the study:**

N/A.

